# Mechanism of baicalein in treatment of castration-resistant prostate cancer based on network pharmacology and cell experiments

**DOI:** 10.3389/fphar.2024.1397703

**Published:** 2024-06-26

**Authors:** Baokai Dou, Yingjie Cui, Qianqian Zhou, Jiawei Fu, Yi Zhou, Xiwu Zhang, Qi Zhang, Jing Zhang

**Affiliations:** ^1^ Department of Pharmacy, Shandong Provincial Hospital Affiliated to Shandong First Medical University, Jinan, Shandong, China; ^2^ Shandong University School of Basic Medical Sciences, Jinan, Shandong, China; ^3^ Department of Urology, Shandong Provincial Hospital Affiliated to Shandong First Medical University, Jinan, Shandong, China

**Keywords:** baicalein, castration-resistant prostate cancer, network pharmacology, cell proliferation, cell cycle

## Abstract

**Objective:**

Baicalein, one of the most abundant flavonoids found in Chinese herb *Scutellaria baicalensis* Georgi, exhibits pharmacological activities against various cancers. However, the precise pharmacological mechanism of baicalein in treating castration-resistant prostate cancer (CRPC) remains elusive. This study aimed to elucidate the potential mechanism of baicalein against CRPC through a combination of network pharmacology and experimental approaches, thereby providing new avenues for research in CRPC treatment.

**Methods:**

The pharmacological and molecular properties of baicalein were obtained using the TCMSP database. Baicalein-related targets were collected from multiple sources including SwissTargetPrediction, PharmMapper and CTD. Targets related to CRPC were acquired from DisGeNET, GeneCards, and CTD. The protein-protein interaction (PPI) was analyzed using STRING 11.5, and Cytoscape 3.7.2 software was utilized to explore the core targets of baicalein on CRPC. GO and KEGG pathway enrichment analysis were performed using DAVID database. Cell experiments were carried out to confirm the validity of the targets.

**Results:**

A total of 131 potential targets of baicalein for the treatment of CRPC were obtained. Among them, TP53, AKT1, ALB, CASP3, and HSP90AA1, etc., were recognized as core targets by Cytoscape 3.7.2. GO function enrichment analysis yielded 926 entries, including 703 biological process (BP) terms, 84 cellular component (CC) terms and 139 molecular function (MF) terms. The KEGG pathway enrichment analysis unveiled 159 signaling pathways, mainly involved in Pathways in cancer, prostate cancer, AGE-RAGE signaling pathway in diabetic complications, TP53 signaling pathway, and PI3K-Akt signaling pathway, etc. Cell experiments confirmed that baicalein may inhibit the proliferation of CRPC cells and induce cell cycle arrest in the G1 phase. This effect could be associated with the TP53/CDK2/cyclin E1 pathway. In addition, the results of CETSA suggest that baicalein may directly bind to TP53.

**Conclusion:**

Based on network pharmacology analysis and cell experiments, we have predicted and validated the potential targets and related pathways of baicalein for CRPC treatment. This comprehensive approach provides a scientific basis for elucidating the molecular mechanism underlying the action of baicalein in CRPC treatment. Furthermore, these findings offer valuable insights and serve as a reference for the research and development of novel anti-CRPC drugs.

## 1 Introduction

Prostate cancer ranks as the second most common male cancer globally (Zhu et al., 2023), with 1,414,259 new cases of prostate cancer (PC) worldwide and 3,75,304 deaths from this type of cancer in 2020 according to the Global Cancer Observatory (Bergengren et al., 2023; Rafikova et al., 2023). Unfortunately, prostate cancer stands as one of the leading causes of cancer-related mortality in men (Rebello et al., 2021). Androgen Deprivation Therapy (ADT) serves is the primary treatment approach for localized advanced or metastatic prostate cancer patients. However, despite undergoing ADT for 12–24 months, most patients progress to develop lethal Castration-resistant Prostate Cancer (CRPC), with a median survival time of merely 14 months (Zhou et al., 2021; Jeon et al., 2023). Therefore, it is of utmost urgency to explore the pathogenesis of CRPC and search for new treatment approaches to improve the survival of CRPC patients.

**FIGURE 1:**
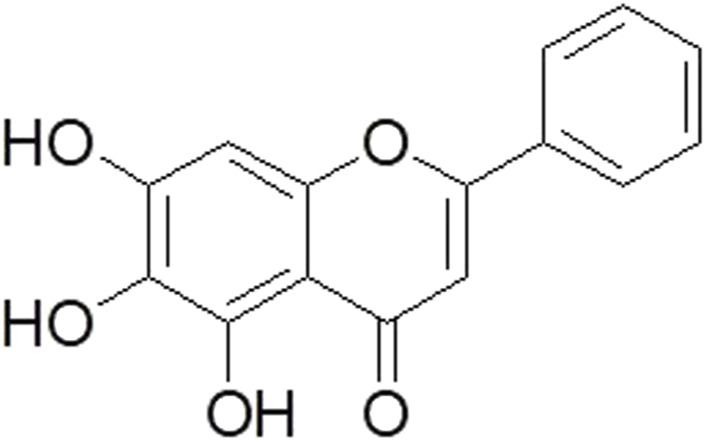
Chemical structure of baicalein.

The characteristic of prostate cancer is androgen dependence, and treatment is mainly based on androgen signal deprivation. However, the molecular mechanisms underlying the occurrence and progression of prostate cancer are intricate and involve the interplay of various factors (Maekawa et al., 2024). These factors encompass tumor suppressor genes, oncogenes, growth factors, signaling molecules, adhesion molecules, angiogenesis, and more (Vietri et al., 2021; Maekawa et al., 2024). *S. baicalensis* Georgi (SBG), commonly known as Scutellaria Radix or Huang-Qin, was first recorded in Shennong’s Herbal Classic in around 200 AD (Xiang et al., 2022). It is one of the most popular herbs in China for its effects of clearing heat and dampness, purging fire, detoxifying, maintaining hemostasis, and preventing miscarriage (Cai et al., 2023a). It is also widely used as medicinal herb in other Asian countries such as Japan, Korea, Mongolia, and Russia, and is cultivated in many European countries (Xiang et al., 2022). In addition, SBG is now listed officially in Chinese Pharmacopoeia (2015), European Pharmacopoeia (EP 9.0), and British Pharmacopoeia (BP 2018) (Xiang et al., 2022; Jang et al., 2023). Baicalein (chemical structure, Figure 1), one of the predominant flavonoids in SBG, has anti-inflammatory, and anti-cardiovascular and cerebrovascular effects (Guo et al., 2021; Dinda et al., 2023; Wang et al., 2023). Typically, the smaller the degree of unsaturation, the more stable the substance will be. Unfortunately, baicalein (C_15_H_10_O_5_) consists of two benzene rings and a pyran ring, its solubility is extremely low and the unsaturation is abnormal (Ω = 11), which is prone to oxidative reactions affecting the stability of the drug and thus affecting the oral bioavailability. But that this lack can be circumvented by chemical modification of the drug or a suitable delivery method, such as HP-β-CD co-lyophilized product, nanocrystal, self-microemulsifying drug delivery system, long-circulating nanoliposomes, and solid dispersionnano-cocrystal strategy (Zhou et al., 2017; Pi et al., 2019).

Recent studies have unveiled baicalein’s potential in combating various tumors. Studies have confirmed that baicalein suppresses the activation of AKT/mTOR pathway by inhibiting the expression of caveolin-1 protein, thereby reducing the proliferation and metastasis of prostate cancer cells (Guo et al., 2015). Ma et al. (2020) demonstrated that baicalein inhibits the proliferation of prostate cancer cells and induces apoptosis by down-regulating the expression level of Ezrin. Baicalein has been found to inhibits the activity of 12-lipoxygenase (12-LOX), which consequently blocks the expression of vascular endothelial growth factor (VEGF) in prostate cancer cells, thereby effectively inhibits tumor angiogenesis (Nie et al., 2006).

In recent decades, many highly cytotoxic chemotherapeutic drugs have been found to be used as first-line drugs in the treatment of various cancers. However, multi-drug resistance caused by prolonged chemotherapy administration limits the successful treatment of cancer patients. Multidrug resistance is mainly attributed to the overexpression of P-glycoprotein (P-gp) in tumor cells, and P-gp efflux pump can significantly reduce the concentration of intracellular chemotherapy drugs (Chen et al., 2021). Baicalein has been reported to modulate the CYP3A subfamily and/or P-gp, reversing P-gp-mediated multi-drug resistance in hepatocellular carcinoma (Li et al., 2018) and overcoming P-gp-mediated resistance (Ferreira et al., 2018). Despite these significant findings, the functional mechanisms of baicalein in the treatment of CRPC is still unknown.

**FIGURE 2:**
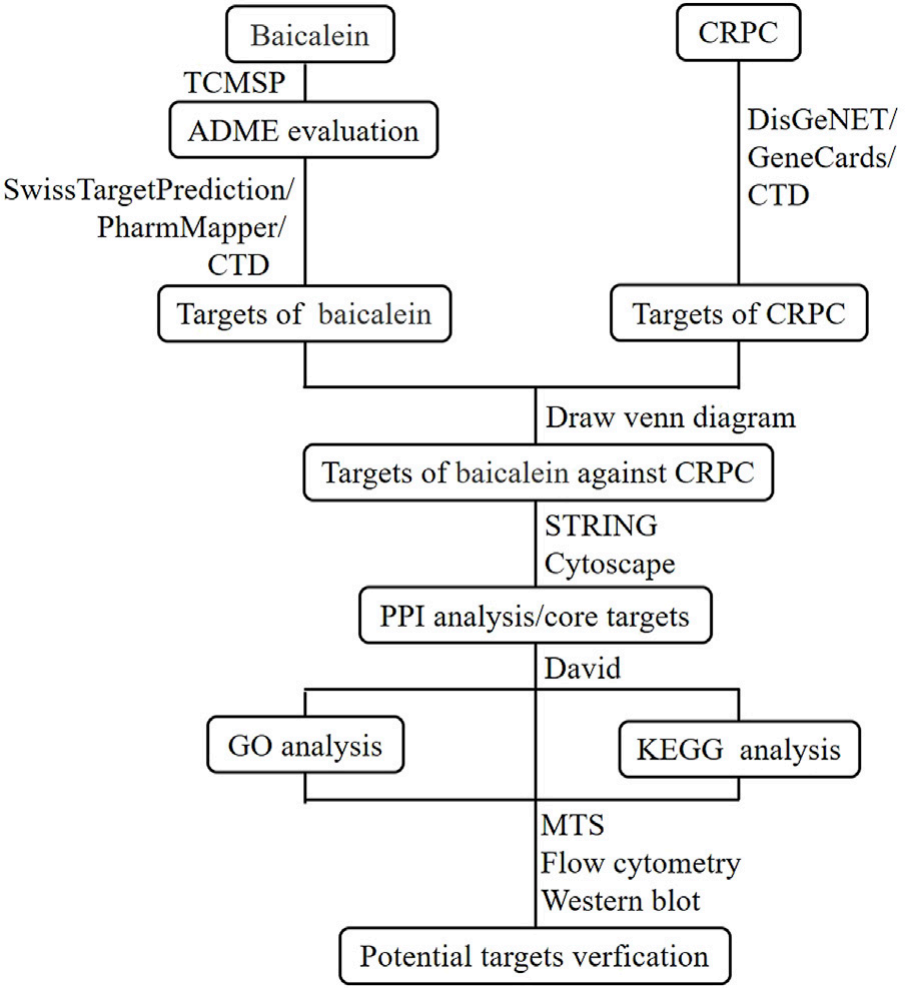
Workflow of baicalein against CRPC.

Network pharmacology is an interdisciplinary field that integrates disciplines such as network science, bioinformatics, computer science, and mathematics (Nogales et al., 2022). It provides foundational theories and research methods for analyzing biological systems’ networks and selecting specific signaling nodes for drug molecule design (Zhao et al., 2023). By employing a systematic approach, network pharmacology investigates drug-target interactions, offering a new framework for innovative drug discovery (Yang et al., 2021). This study utilized network pharmacology methods to predict the potential targets of baicalein against CRPC. Subsequently, cellular experiments were conducted to validate the mechanisms of action of key targets, thereby providing a theoretical basis for the development of anti-CRPC drugs. The flowchart of this study is shown in Figure 2.

## 2 Materials and methods

### 2.1 Collection of ADME characteristics for baicalein using TCMSP

TCMSP (http://tcmspw.com/tcmsp.ph-p) is a unique platform for researching the pharmacological properties of traditional Chinese herbs, can provide Chinese medicine effective component, ADME-related (absorption, distribution, metabolism and excretion) characteristics, drug-target interactions, and networks of disease-related drug targets (Yang et al., 2021). We used “baicalein” as the keyword to search its pharmacological and molecular properties.

### 2.2 Prediction of baicalein-related targets

SwissTargetPrediction (http://www.swisstargetprediction.ch/) database can predict and screen the potential targets of baicalein based on its 2D and 3D structural similarity to known ligands. PharmMapper (http://www.lilab-ecust.cn/pharmmapper/) database can match baicalein’s pharmacophores with internal pharmacophore models in the database to predict its target proteins. PubChem (https://pubchem.ncbi.nlm.nih.gov/) is a public repository of small molecule bioactivity data, and baicalein-related files required for the SwissTargetPrediction and PharmMapper databases can be downloaded. Targets acquired by PharmMapper were converted into corresponding gene names using the Uniprot (https://www.uniprot.org/) platform. CTD (http://ctdbase.org/) integrates a wealth of data on interactions between compounds, genes, functional phenotypes, and diseases. It provides information on environmental exposure factors related to diseases and potential mechanisms of action for drugs. Integrate the predicted drug targets from the SwissTargetPrediction, PharmMapper, and CTD databases and remove duplicate values.

### 2.3 Prediction of CRPC-related targets

The three databases DisGeNET (https://www.disgenet.org/), GeneCards (https://www.genecards.org/) and CTD (http://ctdbase.org/) contain rich, cutting-edge disease-related targets. “Castration-resistant prostate cancer” was used as the search term to obtain the relevant targets of CRPC.

### 2.4 Targets prediction of baicalein anti-CPRC

Potential drug targets and CRC related genes were uploaded to VENNY2.1.0 website (http://bioinfogp.cnb.csic.es/tools/venny/index.html), to determine the cross genes. The common targets represent potential targets of baicalein against CRPC.

### 2.5 Protein-protein interaction (PPI) network construction and core targets screening

The STRING 11.5 (https://STRING 11.5-db.org/) data is one of the richest and most widely used databases for studying protein-protein interactions. The potential targets of baicalein against CRPC were imported into STRING 11.5, the biological species was set as “*Homo sapiens*,” the lowest interaction score was set as medium confidence (0.400), and the rest were default Settings. After obtaining the PPI network from the STRING 11.5 database, Cytoscape 3.7.2 software (https://cytoscape.org/) was used to carry out topology visualization analysis on the PPI network with the topological indicator greater than twice of the median of node Degree. Screening important targets that play a major role in regulating the network. By following these steps, we create a PPI network of potential targets for baicalein’s effect on CRPC and identify key regulatory targets within the network.

### 2.6 GO and KEGG pathway enrichment analysis

Importing core targets into DAVID (https://david.ncifcrf.gov/) Database, select the analyzed organism as “*H. Sapiens*” for GO and KEGG pathway enrichment analysis. The smaller the false discovery rate (FDR), the higher the significance of enrichment. Sort the FDR in ascending order, and use the online tool of bioinformatics to draw GO function enrichment bar charts and KEGG pathway enrichment bubble charts for the top 10. The GO analysis was divided into three parts: biological process (BP), cellular component (CC) and molecular function (MF).

### 2.7 Cell lines, drugs, and reagents

Human CRPC cell line DU145 and C4-2B were purchased from the American Type Culture Collection (ATCC). Baicalein (CAS number: 491-67-8) and dimethyl sulfoxide (DMSO) were purchased from Sigma-Aldrich Trading Co., Ltd. Culture-related reagents, media and fetal bovine serum were purchased from the American company Gibco. Cell culture bottles, culture dishes, etc., were purchased from the American company Corning Costar. MTS Cell Proliferation, Cytotoxicity Assay Kit and Cell Cycle Analysis Kit purchased from Beibo Biological Co., Ltd. TP53 antibody was purchased from Proteintech. Cyclin E1 and CDK2 antibodies were purchased from Abways. Rabbit anti-human GAPDH Antibody: Purchased from Abcam.

### 2.8 Cell culture and grouping

DU145 and C4-2B cells were cultured in RPMI 1640 medium containing 10% fetal bovine serum and incubated in a 5% CO2 atmosphere at 37°C. Baicalein was dissolved in DMSO, and the following groups were set up: blank group, control group (0 μM baicalein), and baicalein-treated groups (10, 20, and 40 μM).

### 2.9 MTS assay for cell viability

The cells were inoculated into 96-well plates at 1,800 cells/well and cultured for 24 h. Subsequent incubations were performed with different concentrations of baicalein. After 24 h, 48 h and 72 h, 10 μL MTS solution was added to each well. The corresponding absorbance was measured after 2 h.

### 2.10 Cell cycle analysis

After treatment with baicalein at various concentrations for 12 h, DU145 and C4-2B cells were collected and cool-fixed at 4°C in 70% ice-cold ethanol overnight. After fixation, the cells were washed twice with cold PBS, and immersed in RNase A solution at 37°C for 30 min, and then incubated with propidium iodide (PI) in a dark place. The cell cycle phases were detected by the flow cytometry system (BD FACSCalibur).

### 2.11 Western blot

After treatment with baicalein at various concentrations for 24 h, DU145 and C4-2B cells were collected. Total protein was extracted using RIPA cell lysis buffer, and the protein concentration was determined using the BCA assay. Twenty micrograms of total protein were loaded onto SDS-PAGE gels, separated by electrophoresis, and then transferred to membranes. The membranes were blocked for 2 h with 5% skim milk and then incubated with primary antibodies against TP53 (1:5000), Cyclin E1 (1:1000), CDK2 (1:1000), and GAPDH (1:3000) at 4 C overnight. After incubation with secondary antibodies at room temperature for 2 h, the blots were visualized using an ECL chemiluminescence detection system.

### 2.12 Cellular thermal shift assay (CETSA)

DU145 and C4-2B cells were inoculated in 10 cm dishes. The next day, the cells were incubated with 100 µM baicalein and solvent control DMSO, respectively, for 2 h at 37°C in an incubator; cells were collected and washed with pre-cooled PBS to remove supernatants later. Then, 1 mL pre-cooled PBS containing 1% protease inhibitor was added for re-suspension. The cell suspensions in PBS were distributed into 8 PCR tubes and heated to indicate temperatures (39, 42, 45, 48, 51, 54, 57°C and 60°C) for 3 min and immediately snap-frozen in liquid nitrogen. Samples were subjected to three freeze-thaw cycles and centrifuged at 12,000 g/min for 20 min. Soluble proteins in the supernatants were transferred into new 1.5 mL microtubes and then analyzed the expression of TP53 by immunoblotting analysis.

### 2.13 Statistical analysis

The data obtained were subjected to statistical analysis using GraphPad Prism 7. The results are presented as mean ± standard error of the mean (Mean ± SEM). For MTS data, a repeated-measures two-way analysis of variance (ANOVA) was used to assess the effects. When comparing multiple groups, a one-way ANOVA was employed for evaluation.

## 3 Results

### 3.1 Pharmacokinetic information of baicalein

Pharmacological and molecular properties of baicalein were obtained by TCMSP (Table 1), including molecular weight (MW), low lipid/water partition coefficient (AlogP), hydrogen bond donors (Hdon), hydrogen bond acceptors (Hacc), oral bioavailability (OB), Caco-2 permeability (Caco-2), blood-brain barrier (BBB), drug likeness (DL), topological polar surface area (TPSA), and number of rotatable bonds (No. of rotb). Lipinski’s criterion, also known as the “rule of five,” is generally considered a drug-likeness test for evaluating the oral availability of a compounds (Ru et al., 2014). A compound is poorly absorbed or permeable when it has more than 5 H-bond donors, more than 10 H-bond acceptors, more than 5 calculated Log P, and the molecular weight higher than 500 Da. In addition, compounds with a topological polar surface area (TPSA) ≤ 140 Å and ≤10 rotatable bonds (nroth) are more likely to have good bioavailability (Han et al., 2019). It is noteworthy that baicalin met all the requirements of Lipinski’s rule of five in terms of oral bioavailability and blood-brain barrier permeability, as shown in Table 1. Furthermore, based on the parameter information and screening criteria of TCMSP database, the OB of baicalein is 30% (≥30%), and the DL value is 0.21, and the DL of effective drugs should be greater than 0.18. Therefore, baicalein demonstrates good drug-likeness.

**TABLE 1:** Pharmacological and molecular properties of baicalein.

Name	MW	AlogP	Hdon	Hacc	OB (%)	Caco-2 (nm/s)	BBB	DL	TPSA (Å)	No. of rotb
baicalein	270.25	2.33	3	5	33.52	0.63	−0.05	0.21	90.89	1

### 3.2 Screening of potential targets of baicalein against CRPC

**FIGURE 3:**
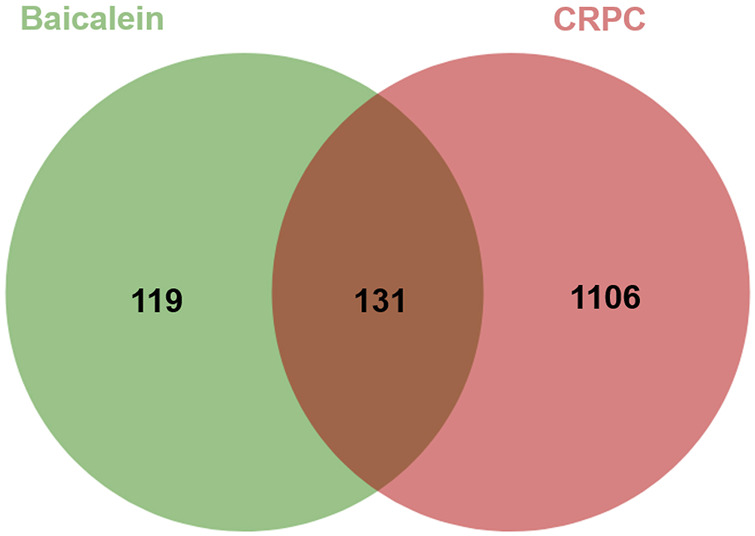
Venn diagram of baicalein targets and CRPC targets. 131 overlapping target proteins between CRPC-related genes and targets of baicalein.

Baicalein’s potential target genes were predicted using the SwissTargetPrediction, PharmMapper, and CTD databases, resulting in a total of 250 potential targets. In parallel, 1,237 potential targets associated with CRPC were obtained from the DisGeNET, GeneCards and CTD databases. Using VENNY2.1 to generate a Venn diagram, 131 intersections were identified as potential candidate targets for baicalein against CRPC (Figure 3).

### 3.3 PPI network analysis and screening of key targets

**FIGURE 4:**
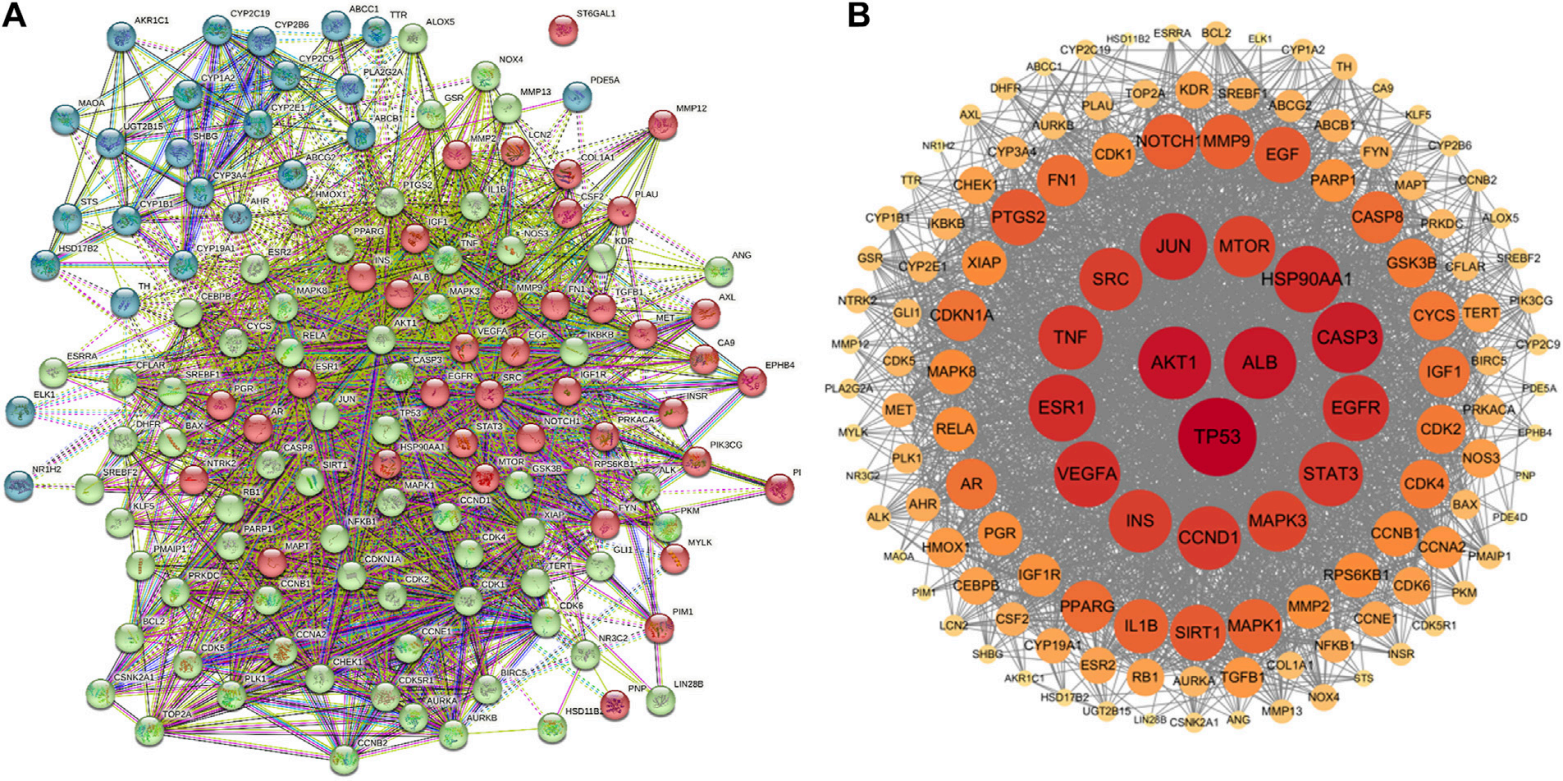
Construction of PPI network. **(A)** PPI network of 131 cross-target. **(B)** The key genes generated by **(A)**. The darker the node color (red), the stronger the connection.

The Protein-Protein Interaction (PPI) network for the cross genes was constructed using STRING 11.5. In the PPI network. A total of 131 genes were input, resulting in 130 nodes with interactions and 2,417 edges, while one node (ST6GAL1) had no connections to others (Figure 4A). The TSV text data from the STRING 11.5 analysis were imported into Cytoscape 3.7.2 software to create the protein interaction network (Figure 4B). A degree greater than twice the median degree value, which was 76, was used as the topological threshold to select core target genes. There were a total of 14 core target genes, including TP53, AKT1, ALB, CASP3, HSP90AA1, JUN, ESR1, EGFR, VEGFA, STAT3, TNF, CCND1, SRC, and INS (Figure 4B).

### 3.4 GO and KEGG pathway enrichment analysis

**FIGURE 5:**
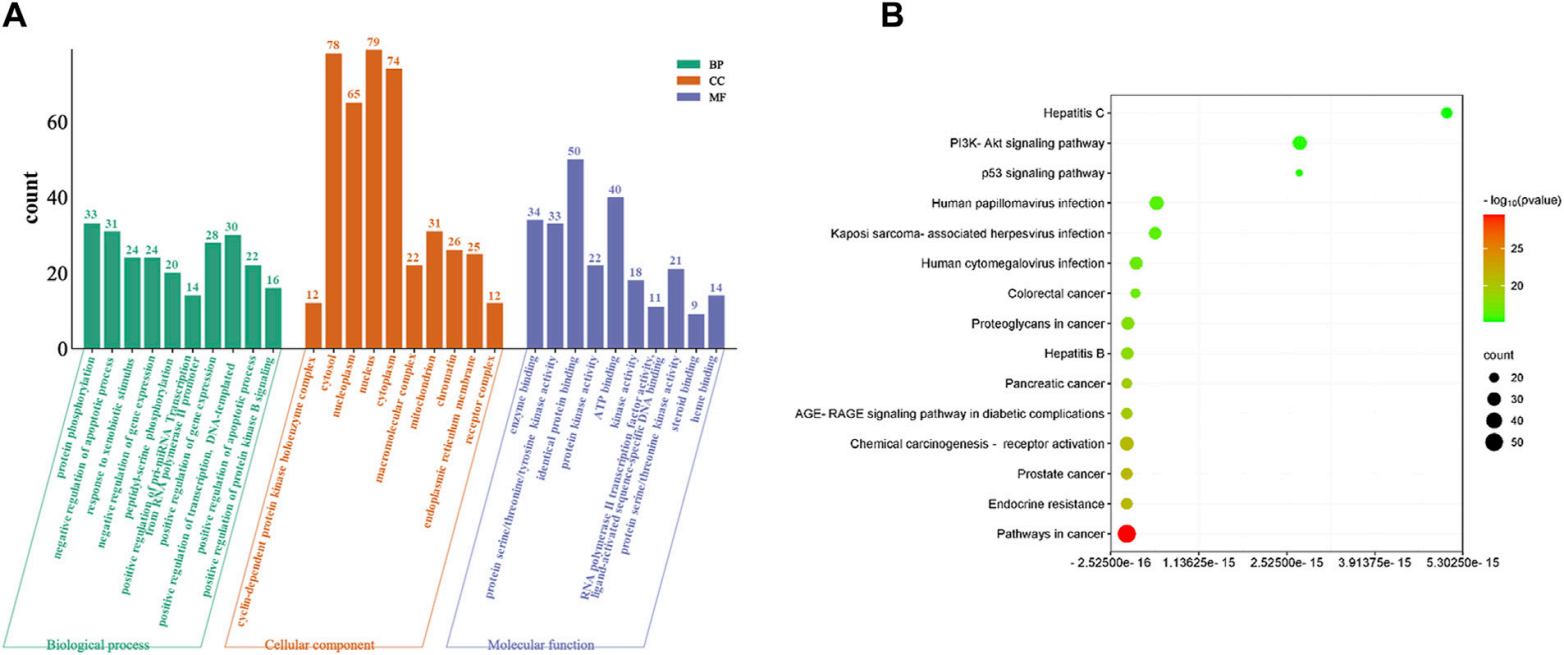
GO and KEGG pathway analysis of intersecting genes. **(A)** GO enrichment analysis, including BP, CC, and MF. **(B)** Bubble diagram of KEGG pathway enrichment.

The functional and pathway enrichment information for 131 cross genes of baicalein against CRPC were analyzed by DAVID database. The results of GO enrichment showed a total of 703 BPs, 84 CCs, and 139 MFs. The top 10 terms (*p* < 0.05) are shown based on *p*-values are displayed (Figure 5A). The main biological processes involved include protein phosphorylation, negative regulation of apoptotic process and response to xenobiotic stimulus, etc. The main cell components include enzyme binding, protein serine/threonine/tyrosine kinase activity and identical protein binding, etc. The results of KEGG pathways analysis indicated that the therapeutic effects of baicalein on CRPC primarily involve 159 signaling pathways. The top 15 pathways (*p* < 0.05), ranked by FDR from lowest to highest, were selected for the generation of an advanced bubble chart (Figure 5B). The identified signaling pathways include Pathways in cancer, prostate cancer, AGE-RAGE signaling pathway in diabetic complications, TP53 signaling pathway, and PI3K-Akt signaling pathway, among others.

The TP53 signaling pathway is a highly attractive novel target for cancer treatment (Kumari et al., 2022). TP53, as a tumor suppressor, can trigger cell cycle arrest, aging, and/or apoptosis on cellular stress, and previous studies have shown that the reactivation of TP53 can be used to treat refractory prostate cancer (Yu et al., 2019; Engeland, 2022). Therefore, in this study, we explored whether baicalein could preserve CRPC cell TP53 function and inhibit tumor growth.

### 3.5 Baicalein inhibits the activity of DU145 and C4-2B cells

**FIGURE 6:**
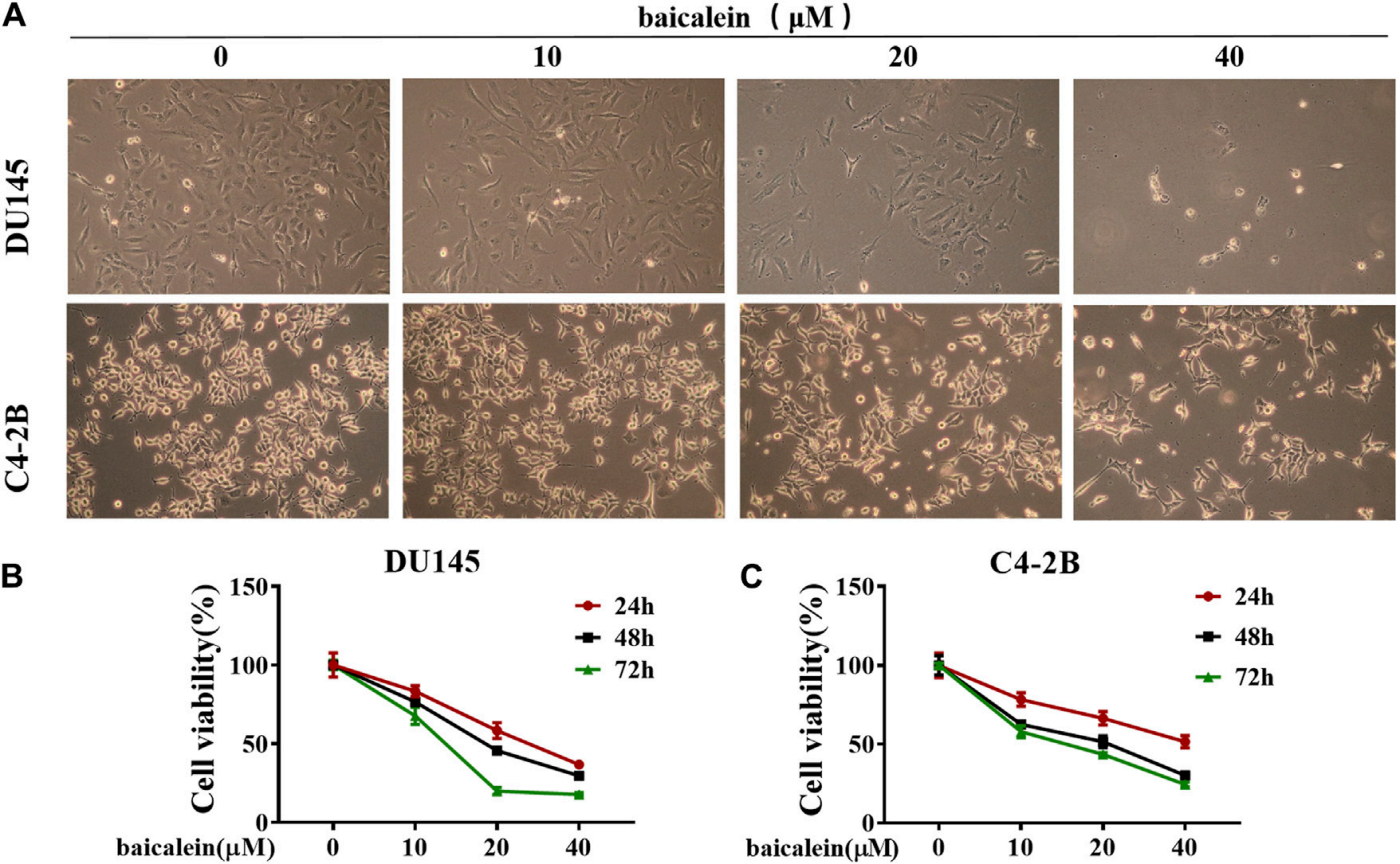
Effect of baicalein on viability of DU145 and C4-2B cells. **(A)** The effects of baicalein on DU145 and C4-2B cells were observed under microscope. **(B,C)** The proliferation of DU145 and C4-2B cells were detected by MTS after treatment with baicalein at different concentrations for 24, 48 and 72 h.

The morphological changes of DU145 and C4-2B cells were observed under a 10× microscope after treatment with baicalein (0, 10, 20, and 40 μM) for 24 h (Figure 6A). Compared to the control group, the number of surviving cells gradually decreased with increasing concentrations of baicalein. The effects of baicalein on the viability of DU145 and C4-2B cells were assessed using the MTS assay after treatment with baicalein (0, 10, 20, and 40 μM) for 24, 48, and 72 h. The results indicate that baicalein significantly inhibited the growth of DU145 and C4-2B cells in a concentration- and time-dependent manner (Figures 6B, C).

### 3.6 Baicalein induces G1 phase cell cycle arrest in DU145 and C4-2B cells

**FIGURE 7:**
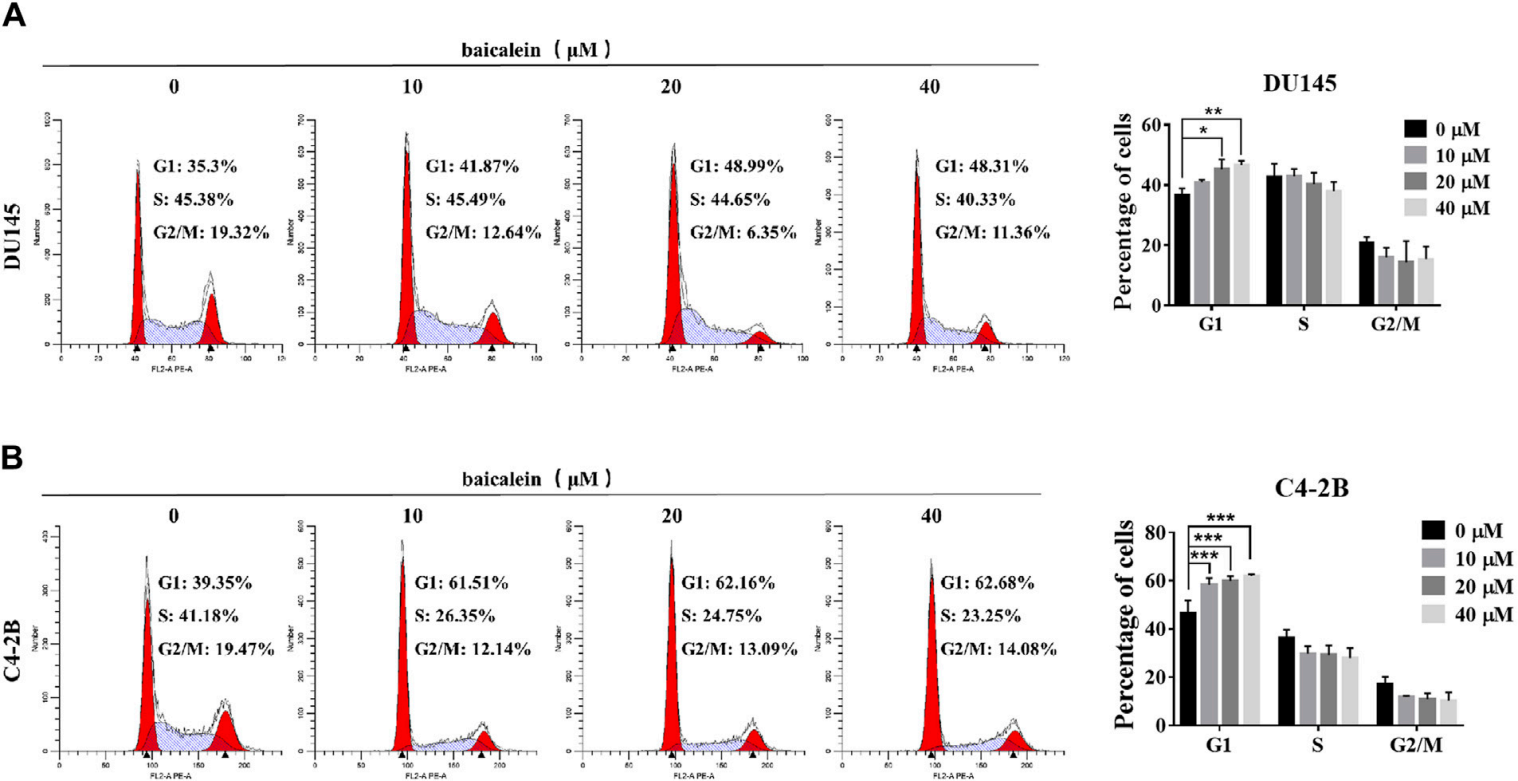
Baicalein induces G1 phase arrest of DU145 and C4-2B cells. **(A, B)** Analyzing the cell cycle arrest after treating with 0, 10, 20, and 40 μM baicalein in DU145 and C4-2B cell lines by flow cytometry. *p < 0.05, **p < 0.01, ***p < 0.001 vs. control.

After treatment with baicalein (0, 10, 20, and 40 μM) for 12 h, the effects of baicalein on the cell cycle of DU145 and C4-2B cells were assessed using flow cytometry. Compared with the control group, baicalein at 20 μM and 40 μM significantly increased the percentage of G1 phase in DU145 cells from 36.51% ± 2.37% to 45.28% ± 3.23% and 46.62% ± 1.47%, respectively (Figure 7A), and baicalein at concentrations of 10 μM, 20 μM, and 40 μM significantly increased the percentage of C4-2B cells in the G1 phase from 44.81% ± 5.07% in the control to 58.35% ± 2.76%, 59.85% ± 2.03%, and 61.94% ± 0.71%, respectively (Figure 7B). These results indicate that baicalein induces G1 phase cell cycle arrest in both DU145 and C4-2B cells.

### 3.7 Effects of baicalein on the expression levels of cell cycle-related proteins

These results indicated that baicalein could significantly inhibit cell growth and induce G1 phase cell cycle arrest. According to the results of network pharmacological analysis, the TP53 signaling pathway is involved in the anti-CRPC mechanism of baicalein. To test this hypothesis, we analyzed the expression levels of cell cycle-related proteins, including TP53, TP53 target gene CDK2, and Cyclin E1.

**FIGURE 8:**
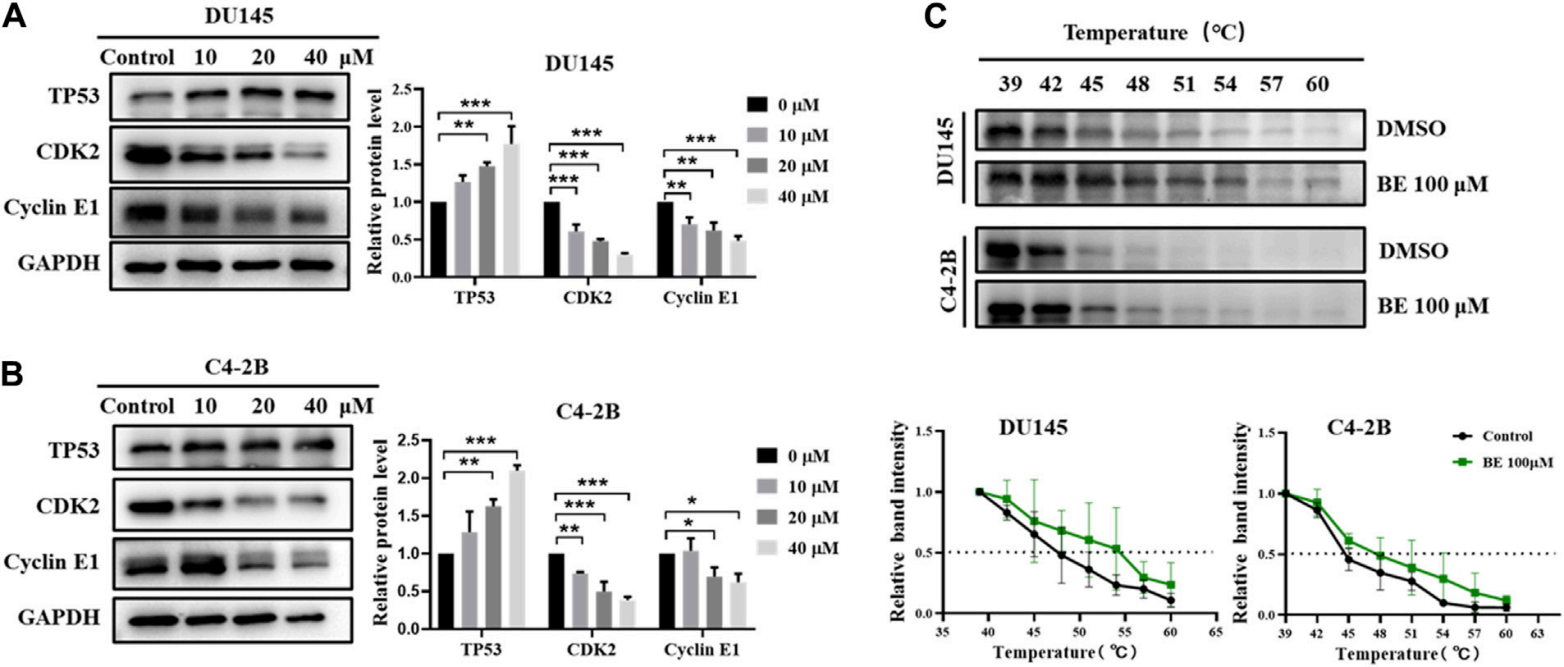
The effect of baicalein on the TP53/CDK2/Cyclin E1 of DU145 and C4-2B cells. **(A, B)** The expression of the cell cycle-related proteins, TP53, CDK2 and Cyclin E1 were identified by Western blotting after treatment of 0, 10, 20, and 40 μM baicalein for 24 h (DU145 and C4-2B). **(C)** CETSA-WB experiment to further confirm the interaction between baicalein and TP53 protein. *p < 0.05, **p < 0.01, ***p < 0.001 vs. control.

The results showed that after treatment with baicalein for 24 h in both DU145 and C4-2B cells, baicalein significantly upregulated the expression of TP53 while downregulating the expression of CDK2 and Cyclin E1 (Figures 8A, B). In addition, to verify that TP53 is indeed the endogenous target of baicalein, cellular thermal shift assay (CETSA) was carried out (Figure 8C). The folding fraction of TP53 decreased with the increasing of temperature and baicalein binding increased the melting temperature (Tm) of TP53 in DU145 and C4-2B cells by 4.4°C ± 0.8°C and 3.5°C ± 1.6°C, respectively, indicating that baicalein may directly binds to TP53. These data suggest that baicalein induces G1 cell cycle arrest and inhibits the proliferation of DU145 and C4-2B cells by targeting TP53.

## 4 Discussion

CRPC, especially metastatic CRPC (mCRPC), is one of the most common malignancies in the world and the leading cause of cancer-related death in men, with reduced life expectancy and a median overall survival rate of less than 2 years (Cai et al., 2023b; Le et al., 2023). As such, overcoming CRPC remains an urgent clinical challenge that needs to be addressed. The mechanism of traditional Chinese medicine in treating diseases is characterized by its multi-target and multi approach. Herbal remedies, extracts and compounds derived from Chinese herbs have been formally applied to prostate cancer in Chinese hospitals, and have been proven to inhibit tumor progression and improve survival rate in patients with metastatic prostate cancer through a variety of mechanisms (Zhang et al., 2022). In this study, big data were used to explore the targets and pathways of baicalein in the treatment of CRPC. By employing network pharmacology and conducting experimental research, our objective is to elucidate the potential molecular mechanisms underlying the anti-cancer effects of baicalein in castration-resistant prostate cancer (CRPC), thereby establishing a solid theoretical foundation for its clinical application and further investigation in this field.

From the ADME-related characteristics of baicalein, it was found that baicalein demonstrates good drug-likeness and warrants further research (Table 1). Combining multiple drug and disease databases, 131 overlapping targets (Figure 3) were initially obtained for constructing PPI networks, with protein-protein interactions present at 130 nodes (Figure 4A). Cytoscape 3.7.2 software was used to screen core targets such as TP53, AKT1, ALB, CASP3 and HSP90AA1, etc. According to the GO enrichment results, baicalein mainly acts in anti-tumor, cell cycle, apoptosis, and so on. And KEGG enrichment analysis revealed that pathways of baicalein against CRPC include AGE-RAGE signaling pathway in diabetic complications, TP53 signaling pathway, and PI3K-Akt signaling pathway, ect. Among them, TP53 inactivation events are very common in mCRPC patients and are associated with a poor prognosis (De Laere et al., 2019; Mateo et al., 2020). Numerous genomic analysis studies have found that a key underlying tumor suppressor, TP53, is frequently mutated in the deadly CRPC, which in turn affects the cell cycle. Zho et al. found that flubendazole, an FDA-approved anthelmintic, is a novel TP53 inducer that exerts anti-proliferative and pro-apoptotic effects in CRPC by blocking the cell cycle and activating iron death (Zhou et al., 2021). In genetic studies in mice, it has also been found that co-deletion of TP53 and PTEN in mouse prostate epithelial cells leads to aggressive prostate cancer and development of CRPC that is newly resistant to androgen deprivation therapy (Shi et al., 2023). Therefore, TP53 can be considered an attractive target for the treatment of CRPC, and there is an urgent need to develop effective treatment strategies for TP53 dysfunction in fatal CRPC.

Further cell experiments showed that baicalein had anti-tumor activity on human CRPC cell lines. More importantly, we observed that baicalein induced G1 cell cycle arrest in CRPC cells. Based on network pharmacological analysis, we investigated the effects of baicalein treatment on cell cycle-related proteins TP53, CDK2, and Cyclin E1. The results showed that after treatment with baicalein for 24 h in both DU145 and C4-2B cells, baicalein significantly upregulated the expression of TP53, downregulated the expression of CDK2 and Cyclin E1, and increased the thermal stability of TP53, suggesting that TP53/CDK2/cyclin E1 pathway may be an effective pathway.

In summary, this study employed an integrated approach combining network pharmacology and cell experiments to elucidate the potential targets and mechanisms underlying baicalein’s efficacy in treating CRPC. The mechanism of action of baicalein against CRPC involves upregulation of TP53 expression and downregulation of CDK2 and Cyclin E1 expression, leading to cell cycle arrest. However, it should be noted that network pharmacology provides a preliminary direction for future research, necessitating further validation through additional experimental studies.

## 5 Conclusion

Collectively, this study comprehensively elucidated the potential mechanism of baicalein in the treatment of CRPC through an integrated approach involving network pharmacology and cell experiments. Our findings demonstrate that baicalein exhibits significant anti-proliferative effects and induces cell cycle arrest in CRPC cells by modulating the TP53/CDK2/cyclin E1 pathway. These results provide compelling evidence for the therapeutic application of baicalein in treating CRPC and establish a solid foundation for further exploration of its drug potential.

## Data Availability

The raw data supporting the conclusion of this article will be made available by the authors, without undue reservation.
